# Expression of TRPC6 channels in human epithelial breast cancer cells

**DOI:** 10.1186/1471-2407-8-125

**Published:** 2008-05-02

**Authors:** Arnaud Guilbert, Isabelle Dhennin-Duthille, Yassine EL Hiani, Nathalie Haren, Hafida Khorsi, Henri Sevestre, Ahmed Ahidouch, Halima Ouadid-Ahidouch

**Affiliations:** 1 Laboratoire de Physiologie Cellulaire et Moléculaire, JE « Canaux ioniques dans le cancer du sein », Faculté des Sciences, Université de Picardie Jules Verne, 33 Rue St Leu 80039, Amiens, France; 2 Dysrégulations Métaboliques Acquises et Génétiques, Faculté de Médecine, Université de Picardie Jules Verne, 3 rue des Louvels, 80036, Amiens, France; 3 Service d Anatomie Pathologique, CHU Nord, Amiens, France; 4 Laboratoire de Physiologie Animale, Faculté des Sciences, Université Ibn-Zohr, Agadir, Morocco

## Abstract

**Background:**

TRP channels have been shown to be involved in tumour generation and malignant growth. However, the expression of these channels in breast cancer remains unclear. Here we studied the expression and function of endogenous TRPC6 channels in a breast cancer cell line (MCF-7), a human breast cancer epithelial primary culture (hBCE) and in normal and tumour breast tissues.

**Methods:**

Molecular (Western blot and RT-PCR), and immunohistochemical techniques were used to investigate TRPC6 expression. To investigate the channel activity in both MCF-7 cells and hBCE we used electrophysiological technique (whole cell patch clamp configuration).

**Results:**

A non selective cationic current was activated by the oleoyl-2-acetyl-sn-glycerol (OAG) in both hBCE and MCF-7 cells. OAG-inward current was inhibited by 2-APB, SK&F 96365 and La^3+^. TRPC6, but not TRPM7, was expressed both in hBCE and in MCF-7 cells. TRPC3 was only expressed in hBCE. Clinically, TRPC6 mRNA and protein were elevated in breast carcinoma specimens in comparison to normal breast tissue. Furthermore, we found that the overexpression of TRPC6 protein levels were not correlated with tumour grades, estrogen receptor expression or lymph node positive tumours.

**Conclusion:**

Our results indicate that TRPC6 channels are strongly expressed and functional in breast cancer epithelial cells. Moreover, the overexpression of these channels appears without any correlation with tumour grade, ER expression and lymph node metastasis. Our findings support the idea that TRPC6 may have a role in breast carcinogenesis.

## Background

Breast cancer has the highest incidence rate for cancer in women in industrialized countries. Statistically, it is estimated that one woman out of ten will develop breast cancer at some point in her life. Evidence is accumulating for the role of ion channels in the development of cancer. The most studied ion channels in breast cancer are firstly K^+ ^channels, which are involved in proliferation, cell cycle progression and migration [1-4], and secondly Na^+ ^channels which correlate with invasion [5-7]. Apart from the role of intracellular calcium in MCF-7 apoptosis [8], little is known of Ca^2+ ^homeostasis in breast cancer cells. The first study reported by Strobl et al., [9], suggested that Ca^2+ ^is necessary for the cell cycle progression in breast cancer cells. Moreover, the early findings of Sergeev indicate that voltage-insensitive channels and Ca^2+ ^endoplasmic reticulum stores are the principal pathways for Ca^2+ ^entry in MCF-7 breast cancer cell line [8,10]. Recently, Guo et al., [11], have reported that the inhibition of a voltage-independent calcium channel induced growth inhibition and apoptosis in breast cancer cells. However, until now, the channel types involved in this cationic current have remained unknown. Recent findings demonstrate that the expression and/or activity of the TRP superfamily, have been reported to be involved in colorectal, colon, thyroid, breast, ovarian, pancreatic and prostate cancer [12,13]. Among these, prostate cancer has been the most studied [12,14-17]. Indeed, TRPV6 is strongly expressed in advanced prostate cancer, with no expression either in healthy or in benign prostate tissues [17]. Moreover, the TRPV6 expression correlates with the Gleason score [18], and with aggressiveness [14,16,18]. Another type of TRP: TRPM8 was detected at high levels in both benign prostate hyperplasia and in prostate carcinoma cells, as well as at low levels in normal (non-carcinoma) prostate epithelial cells. According to these data, TRPM8 has recently been proposed as a molecular target [13,19] and TRPV6 as a general marker for neoplasma [15,20].

The TRPC subfamily, TRPC1 TRPC7, has mostly been implicated in regulation by G-proteins and metabolites of phosphoinositide hydrolysis. TRPC6 channels, known to be activated by the phospholipase C (PLC) product, Diacylglycerol (DAG) [21-24], is abundantly expressed and plays an important role in lung tissues and in different smooth muscle cell types [22,25-27].

TRPC6 was suggested as being the molecular correlate to the α1-adrenoceptor-activated non-selective cation channel in vascular smooth muscle cells [24,28]. Moreover, TRPC6 is also involved in some smooth and cardiac muscle pathologies [27,29,30]. TRPC6 is also expressed in epithelial human prostatic cancer cells and the Ca^2+^-entry via this channel mediates the activation of calcineurin, which in turn induces proliferation via its downstream NFAT (nuclear factor of activated T-cells) transcription factors, which are necessary and sufficient for the induction of prostatic cancer cell proliferation [31].

Previously, we have reported that TRPC6 is expressed in MCF-7 [32]. However, until now, little is known about the relative expression of TRPC6 in normal and cancerous breast cells.

The aim of this study is, on the one hand, to compare the expression of TRPC6 in normal and cancerous human breast tissues. On the other hand, we have sought to investigate the role of TRPC6 by using electrophysiological and molecular techniques. To do this, we used breast tissue specimens, primary cultures of human breast cancer epithelial (hBCE) cells and MCF-7 cell line.

## Methods

### Cell culture

MCF-7 cells were cultured in Eagle's Minimum Essential Medium (EMEM), supplemented with 5% foetal calf serum (FCS), 2 mM L-glutamine, and 0.06 % Hepes buffer, and maintained at 37°C in a humid atmosphere of 5% CO_2 _in air.

### Immunohistochemistry

Normal and cancerous breast tissues were obtained from fresh surgical specimens. Surgical consent forms (approved by the University Hospital of Amiens) were signed by the patients before surgery to allow the use of a portion of the tissue for research purposes.

49 normal and cancer human breast specimens were obtained from women having undergone operations at the Amiens Hospital, France. Normal breast samples were taken at a distance from the tumour. Regarding tumour grade in the 49 invasive ductal breast carcinomas, 15 were of Grade I (well-differentiated), 19 were of Grade II (moderately-differentiated) and 15 were of Grade III (poorly-differentiated). On diagnosis, 23 tumours presented lymph-node metastasis.

Immunohistochemical studies were performed using the indirect immuno-peroxidase staining technique on the paraffin-embedded material with a Ventana ES automatic analyzer (Ventana Medical Systems) and with a hematoxylin counterstain. Briefly, after blocking the endogenous peroxidase by the I-View Inhibitor (Ventana), sections were stained with an anti-TRPC6 antibody (Chemicon, 1/300) for 32 min, washed, incubated with biotinylated anti-rabbit IgG (I-View Biotin Ig, Ventana) for 8 min, washed and exposed to streptavidine-peroxidase complex (I-View SA-HRP, Ventana) for 8 min. DAB/H2O2 was used as chromogen and the slides were then examined under optical microscopy. Micrograph acquisitions were performed by a camera connected to a Zeiss microscope equipped with 20 × 0.85 objective lens.

Immunostaining levels in the tumour tissue were determined by subjective visual scoring of the brown stain, and compared to the normal tissue. Scoring levels were: 0 = absence of staining; 1 = weak staining intensity (equal to normal tissue); 2 = moderate; 3 = strong staining intensity. For the quantitative analysis, we report the percentage of cases presenting an overexpression of TRPC6 (scores 2 and 3).

Peptide blocking was performed as follows. The TRPC6 peptide (1 μg, Chemicon) was incubated with the primary antibody (1 μg) for one hour at room temperature. The complex was then applied to the sections in place of the diluted primary antibody and staining was completed as already described.

### Primary culture

Portions of human breast cancerous tissues were placed in transport medium and desegregated immediately or after storage at 4°C for less than 6 h. The transport medium contained RPMI 1640 medium, 100 U/ml penicillin, 0.1 mg/ml streptomycin, 2 mM glutamine, 10% FBS and 0.010 mg/ml insulin. Adipose or gross and necrotic materials were removed and the tissue minced using a scalpel in phosphate buffer solution (PBS) pH 7.4 under sterile conditions. Cancerous tissues were digested in transport medium containing 1 mg/ml collagenase type I (Sigma, France) and 100 U/ml hyaluronidase (Sigma, France) overnight at 37°C. When digestion was completed, tissue suspensions were centrifuged at 1000 rpm for 5 min and the pellets resuspended in sterile PBS pH 7.4. The dispersed cell suspensions were centrifuged at 1000 rpm for 5 min and pellets resuspended in 20% FBS growth medium (RPMI 1640 medium, 100 U/ml penicillin, 0.1 mg/ml streptomycin, 2 mM glutamine, 0.005 mg/ml insulin, 5 ng/ml epidermal growth factor (EGF), 0.5 μg/ml hydrocortisone, 5 μg/ml transferrin, 0.1 μM isoproterenol, 0.01 μM ethanolamine, 0.01 μM *o*-phosphoetanolamine) and seeded in culture flasks (Nunc, Poly Labo, Strasbourg, France) and kept at 37°C in a humidified incubator in a 95% air 5% CO2 atmosphere. Each sample was analyzed by immuno-fluorescence staining to verify the pan-cytokeratin expression, which is an epithelial marker.

We used specimens from invasive ductal breast carcinomas and clinical tumour (Grade II), from patients having undergone a mastectomy. None of the patients had a history of chemotherapy and/or anti-estrogens therapy. The absence of normal epithelial cells was confirmed by independent histologic and anatomopathologic analysis.

### Electrophysiology

For electrophysiological analysis, cells were cultured in 35 mm Petri dishes at a density of 5.10^4 ^cells 2 days before patch clamp experiments. Currents were recorded in voltage-clamp mode, using an Axopatch 200 B patch-clamp amplifier (Molecular devices) and a Digidata 1200 interface (Molecular device). PClamp software (v. 6.03, Molecular device) was used to control voltage, as well as to acquire and analyze data. The whole-cell mode of the patch-clamp technique was used with 3–5 MΩ resistance borosilicate fire-polished pipettes (Hirschmann^®^, Laborgerate). Seal resistance was typically in the 1–5 GΩ range. Whole cell currents were allowed to stabilize for 5 min before being measured. Cells were allowed to settle in Petri dishes placed at the opening of a 250 μm-inner diameter capillary for extra-cellular perfusions. The cell under investigation was continuously superfused with control or test solutions. All electrophysiological experiments were performed at room temperature.

### Total RNA isolation and reverse transcription of RNA

Total RNA from MCF-7 cells and primary culture cells was extracted by the Trizol-phenol-chloroforme (Sigma Aldrich) procedure, including DNAse I treatment (0.2 U/μl, 30 min at 37°C, Promega). Total RNA was then reverse-transcribed into cDNA using oligodT primers and SuperScript™ II Reverse Transcriptase (Invitrogen).

RNA isolation of normal and tumour tissues was performed using the RNAeasy Mini Kit (Qiagen). Pieces of tissue (20 mg) were placed in a lysis buffer and homogenized using a Polytron homogenizer (PRO-200, Fisher Bioblock Scientific), and total RNA was isolated according to the manufacturer s standard protocols and used (1 μg) for first-strand cDNA synthesis with oligodT primers and MultiScribeTM Reverse Transcriptase (Applied Biosystems).

### Qualitative and semi-quantitative PCR

Sense and antisense PCR primers specific to TRPC3, TRPC6, TRPC7 channels, β-actin and cytokeratin 19 (CK19) were used (see Table 1: primers for PCR experiments). PCR reactions were carried out on a iCycler thermal cycler (Biorad) using Taq DNA polymerase (Invitrogen) using the following parameters: denaturation at 94°C for 30 s, annealing at 58°C for 30 s, and extension at 72°C for 40 s. A total of 30 cycles for actin and 40 cycles for the other primers were performed, followed by a final extension at 72°C for 5 min. PCR products were analyzed by electrophoresis with 1.5% agarose gel and visualized by ethidium bromide staining.

**Table 1:** Primers for PCR experiments

Gene	Accession n°	Primer	Sequence (5-3)	Predicted Size, bp
hTRPC3	NM003305	Sense	GGAAAAACATTACCTCCACCTTTCA	
		Antisense	CTCAGTTGCTTGGCTCTTGTCTTCC	383 pb
hTRPC6	NM 004621	Sense	GAACTTAGCAATGAACTGGCAGT	625 pb for TRPC6
		Antisense	CATATCATGCCTATTACCCAGGA	277 pb for TRPC6γ
hTRPC7	NM 020389	Sense	GTCCGAATGCAAGGAAATCT	
		Antisense	TGGGTTGTATTTGGCACCTC	477 pb
hβ-actin	NM 001101	Sense	CAGAGCAAGAGAGGCATCCT	
		Antisense	ACGTACATGGCTGGGGTG	210 pb
hCK19	NM 002276	Sense	GATTGCCACCTACCGC	
		Antisense	CCATCCCTCTACCCAG	136 pb

For the semi-quantitative experiments, 40 cycles, 25 cycles and 35 cycles were performed for TRPC6, β-actin and CK19 respectively. After agarose gel electrophoresis, PCR products were quantified using Quantity One software (Biorad) and expressed as the ratio of TRPC6 on β-actin or CK19 referent genes. CK19 has been shown to be specific to breast epithelial cells, both normal and malignant [33].

### Western Blotting

Prostate human cancer cell line (LNCaP), MCF-7 cells and primary culture cells were lysed for 30 min on ice in RIPA buffer (1% triton ×100, 1% Na deoxycholate, 150 mM NaCl, 10 mM PO4Na_2_/K pH 7.2) supplemented with Sigma P8340 inhibitor cocktail, 2 mM EDTA and 5 mM Na orthovanadate. After centrifugation at 13000 rpm, the proteins in the supernatant were quantified using the BCA method (Biorad).

Breast tissue proteins were extracted using the WCE buffer (Whole Cell Extract : 150 mM NaCl, 50 mM Tris HCl pH7.5, 1% NP40) supplemented with Sigma P8340 inhibitors cocktail, 0.1% SDS and 1 mM Na orthovanadate. After 1 hour in lysis buffer at 4°C, tissues were homogenized using a Polytron homogenizer (PRO-200, Fisher Bioblock Scientific) and frozen 20 min at -80°C. After centrifugation at 13000 rpm, the proteins in the supernatant were quantified using the BCA method (Biorad).

Equal amounts of each protein sample (15–20 μg) were separated by electrophoresis on SDS-PAGE and blotted onto nitrocellulose membrane (Amersham). Blots were incubated with antibodies raised against TRPC6 (1/300, Chemicon) or β-actin (1/1000, Santa Cruz) and developed with the enhanced chemiluminescence system (ECL, Amersham) using specific peroxidase-conjugated anti-IgG secondary antibodies. Peptide blocking was performed as described in the immunohistochemistry section.

### Solutions

External and internal solutions had the following compositions (in mM): External: NaCl 140, KCl 5, MgCl_2 _2, CaCl_2 _2, HEPES 10 and glucose 5 at pH 7.4 (NaOH). Internal: CsCl 140, CaCl_2 _5, ATP-K_2 _1, HEPES 10, EGTA 10, MgCl_2 _2, at pH 7.2 (CsOH). The [Ca^2+^]i was clamped to 85 nM and calculated with WebMaxC v2.1 (please see Availability & requirements section below).

Extracellular and intracellular osmolarity measured with a freezing-point depression were 300 mOs and 292 mOs respectively. In order to completely block K^+ ^channels, we added TEA at 5 mM to the extracellular medium. 2-APB, SK&F 96365 and OAG (Sigma, France) were dissolved in DMSO. Final concentrations were obtained by appropriate dilution in an external control solution. The final DMSO concentration was < 0.1%.

### Statistical analysis

Results were expressed as mean ± S.E. The Student s t test was used to compare the relative TRPC6 transcripts in normal and cancer tissues. P < 0.05 was considered as significant. Immunostaining in the epithelial compartment of tumour tissues compared to normal tissues was scored visually as equal expression or overexpression of TRPC6. χ^2 ^tests were used in GraphPad Software to estimate the correlation between TRPC6 overexpression and clinical characteristics of the carcinoma tissues. A correlation was considered significant when P < 0.05.

## Results

### OAG induced a non-selective cationic current in the MCF-7 breast cell line

**Figure 1:**
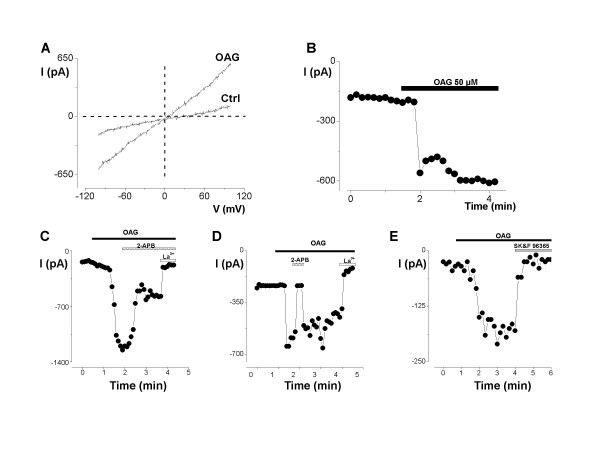
**Electrophysiological and Pharmacological characterisation of the OAG-induced current in MCF-7 cell line**. A) Typical current trace recorded during the current induced by 50 μM OAG. The holding potential was -40 mV and 400 ms voltage ramps were applied from -100 mV to +100 mV. The reversal potential (E_rev_) of the currents recorded was about 0 mV. B) Time course development of OAG-inward currents (measured at -100 mV) in a representative MCF-7 cell bathed in external control medium. C-D) 2-APB (50 and 100 μM) and La^3+ ^(100 μM) effects on time course of OAG-induced current recorded at -100 mV. E) The extracellular perfusion of SK&F 96365 (10 μM) completely inhibited the current induced by OAG (50 μM).

We have previously reported that TRPC6 is the detectable member of the store-independent TRPC channels expressed in breast cancer cells [32]. Recent studies have revealed that the permeant DAG analogue, OAG can stimulate an inward current in cancerous epithelial cells [31]. We recorded OAG-induced channel activity in MCF-7 cells using the whole cell patch clamp configuration, replacing K^+ ^with Cs^+ ^to block K^+ ^channels and using a high [Ca^2+^]i to prevent the passive intracellular Ca^2+ ^store depletion that could lead to activation of store operated channels (SOC, I_soc _current) (Fig. 1). An application of 50 μM of OAG to the bath induced a linear current in MCF-7 cells. The current/voltage dependence of this OAG-induced current displayed both inward and outward currents (Fig. 1A). This current revealed a pattern of a non-selective cation current (Fig. 1A) with a reversal potential close to 0 mV (7.2 ± 1.4, n = 12). The average density of OAG-induced current measured at -100 mV varied between -25 and -12.5 pA/pF with a mean of -18.3 ± 2.7 pA/pF (n = 10). The time course for OAG-induced current at -100 mV revealed an inward current which reached a peak in about 3 min (Fig. 1B).

### Pharmacological properties of the OAG-induced current

A number of pharmacological agents have been used to characterize DAG-induced currents or Ca^2+ ^entry pathways in a variety of cell types [34]. Here we show that 2-APB reduced and completely inhibited the OAG-induced current in MCF-7 cells when used at 50 μM and 100 μM respectively (Fig. 1CD, n = 4), and this effect was totally reversed after washout (Fig. 1D). Moreover, the OAG-sensitive current was also completely inhibited by 100 μM La^3+ ^(Fig. 1CD, n = 6) and by 10 μM SK&F 96365 (Fig. 1E, n = 3).

### OAG also induced a cationic current in primary epithelial breast human cells (hBCE)

**Figure 2:**
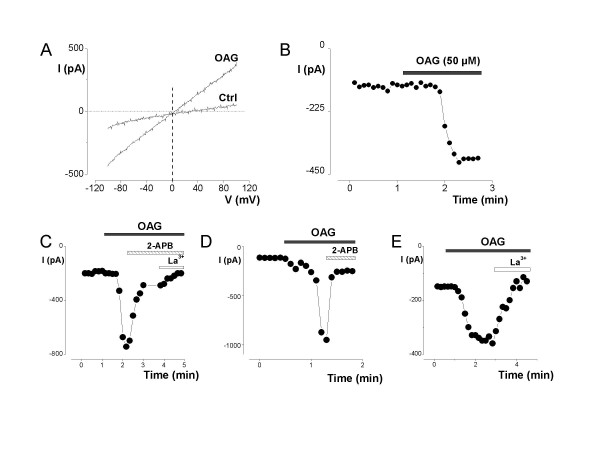
**OAG mediated an inward cationic current in primary hBCE**. A) Current-potential relationship in the absence and presenceof OAG (50 μM). The holding potential was -40 mV and 400 ms voltage ramps were applied from -100 mV to +100 mV. B) Time course of the inward whole-cell membrane current activated by OAG (50 μM). C-E) Effects of the common cationic channels inhibitors, 2-APB 50 μM (C), 100 μM (D), and 100 μM La^3+ ^(C, E) on the amplitude of OAG-induced inward current measured at -100 mV.

A typical OAG-induced current could also be recorded in hBCE cells. A representative example is shown in Fig. 2AB. The average density of the OAG-induced current measured at -100 mV varied between -50 and -20.6 pA/pF with a mean of -34.3 ± 4.6 pA/pF (n = 7). The current that we recorded had a E_rev _about 0 mV (6.0 ± 2.4 mV, (n = 4)) and was again reduced by 50 μM 2-APB (Fig. 2C, n = 6) or completely blocked by 100 μM 2-APB (Fig. 2D, n = 6) and by La^3+ ^(100 μM, n = 6) (Fig. 2CE).

### Expression of OAG-gated TRP channels in human breast cancer epithelial cells

**Figure 3:**
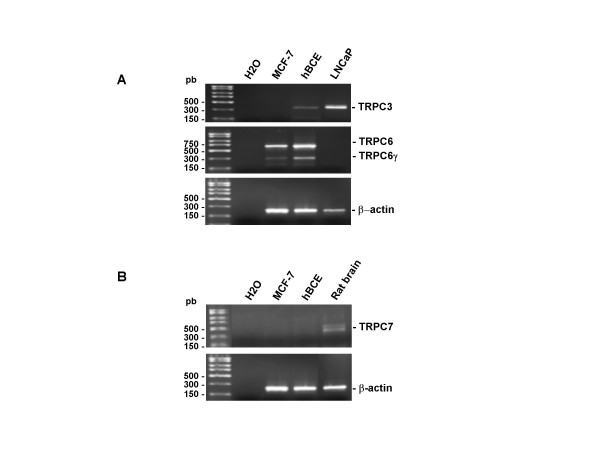
**Analysis of TRPC3, TRPC6 and TRPC7 mRNA expression in MCF-7 and hBCE cells**. RT-PCR analysis of the expression of human TRPC3, TRPC6 (A), and TRPC7 (B) transcripts in hBCE and MCF-7 cells. The PCR products were obtained using the primers described in Table 1. Rat brain and the human prostate cancer epithelial cell line (LNCaP) were used as positive controls for the detection of TRPC7, and TRPC3 respectively. LNCaP was also used as negative control for TRPC6.

TRPC3, TRPC6 and TRPC7 are reported to be directly gated by DAG and OAG [23,35]. Therefore, in order to determine potential candidates for the OAG-coupled cationic channel(s) in hBCE, we used RT-PCR to analyse the expression of the specific transcripts for the human isoforms of these DAG-gated TRP members in these cells. As expected, MCF-7 cells expressed only TRPC6 while hBCE expressed both TRPC6 and TRPC3 transcripts (Fig. 3A), whereas TRPC7 was undetectable (Fig. 3B) in either cell. The prostate cancer cell line (LNCaP) was used as a positive control for TRPC3 and a negative one for TRPC6. Indeed, it has been reported that LNCaP cells express TRPC3 but not TRPC6 [36]. Moreover, both MCF-7 and hBCE cells express a TRPC6γ splice variant (Fig. 3A).

### TRPC6 is expressed in breast tumour tissues

**Figure 4:**
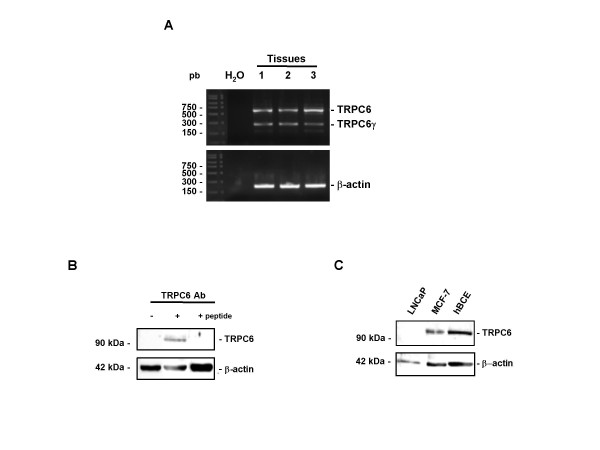
**Expression of TRPC6 in human breast cancer**. A) Expression pattern of TRPC6 mRNA. The PCR products were observed in 3 human tumour breast tissues. A representative Western blot of TRPC6 (around 97 kDa) was performed on breast tumour tissues (B), MCF-7 and hBCE cells (C). TRPC6 Ab : TRPC6 antibody; (-) incubation of tumour tissue protein lysates with only the second antibody; (**+ peptide**) protein lysates are incubated with a mixture of TRPC6 antibody and TRPC6 peptide. Protein lysates from LNCaP cells were used as negative control (C).

No data are available on the expression of TRPC6 in human carcinoma tissue. Fig. 4 shows the expression of the transcripts for the TRPC6 in human breast tissue (Fig. 4A). Moreover, we also observed the expression of the TRPC6γ splice variant. Using Western blotting, we found that the TRPC6 proteins were expressed in breast carcinoma (Fig. 4B). There was no band when we omitted the primary antibody or when we blocked the TRPC6 antibody with the TRPC6 peptide (Fig. 4B). Moreover, TRPC6 is also expressed at a protein level both in MCF-7 cells and in hBCE (Fig. 4C). Again, there was no band when we used lysate from LNCaP (Fig. 4C).

### Overexpression of TRPC6 in breast adenocarcinoma

**Figure 5:**
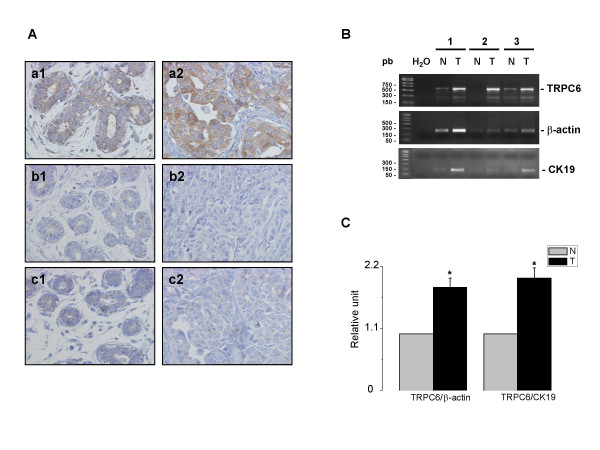
**Overexpression of TRPC6 in breast adenocarcinoma**. A) Normal (a1) and tumour (a2) breast tissues show a respectively weaker and a stronger staining when incubated with TRPC6 antibody. (b1) and (b2) are the same field showing no staining when the primary antibody was omitted. TRPC6 staining of normal (c1) and tumour (c2) breast tissue was blocked by the preincubation of TRPC6 peptide with the primary antibody. (magnification, × 200). B) TRPC6 transcripts were detected in breast tissues (N = normal, T = tumour) by semi-quantitative PCR using TRPC6, β-actin, or CK19 primers. C) Relative TRPC6 expression in breast cancer tissues. TRPC6 mRNA was quantified using Quantity One software and normalized to β-actin or CK19 expression. Data are presented as the average of duplicate experiments on three distinct patients (* P < 0.05).

Immunohistochemical analysis was performed on 49 normal tissues and ductal breast carcinomas. Normal breast specimens were obtained from mammectomy specimens, at a distance from the tumour. High expression of TRPC6 was detected in tissue of breast cancer and small or negative expression was detected in normal tissue. The frequency of TRPC6 expression in breast cancer averaged 73.4% (36/49) of the cases studied. Fig. 5A shows representative positive expression of neoplastic tissue of breast cancer. The staining of breast adenocarcinoma (a2) demonstrated a stronger positive reaction than its normal counterpart (a1). There was no or little staining when the primary antibody was omitted (b1, b2), or after preincubation of TRPC6 peptide with the primary antibody (c1, c2).

Using semi-quantitative PCR, we analyzed TRPC6 mRNA expression in tumour and normal tissues from 3 individual patients. As shown in Fig. 5B &5C, TRPC6 mRNA expression was generally higher in the tumour tissues after normalization both with CK19 or β-actin (P < 0.05).

We next compared the expression of TRPC6 with LNM, estrogen receptor (ER) expression, and tumour grade. A quantitative analysis, using *χ*^2 ^statistical test, of the results obtained is reported in Table 2. The TRPC6 protein expression was similar in tumour tissues associated (78%, n = 23) or not associated (69%, n = 26) with LNM. Non significant differences in the TRPC6 expression were also found with estrogen receptor expression. Regarding TRPC6 channel protein expression with tumour grade, we found that TRPC6 was expressed in 73.3% and 68.4% of grade I and grade II respectively, and increased to 80% in grade III.

**Table 2:** Comparison of TRPC6 expression to tumour characteristics on 49 patients using χ2 analysis.

	TRPC6 overexpression	*n*	*X*^2^
LNM status			
LNM	78.3%	23	
no LNM	69.2%	26	0.4749
ER status			
ER+	70.9%	31	
ER-	81.8%	11	0.5180
Tumor grade			
1	73.3%	15	
2	68.4%	19	0.4473
3	80%	15	0.4473

Absence of significant statistical correlation between TRPC6 overexpression and tumour characteristics (LNM: lymph node metastasis; ER: estrogen receptor).

## Discussion

Many recent works report the involvement of TRP channels in cancer. Our results point towards an aberrant expression of TRPC6 channels in breast cancer. This study shows that TRPC6 is expressed in both the MCF-7 breast cancer cell line and in the primary cultures of breast cancer epithelial cells. TRPC6 appears to be functional both in MCF-7 and in hBCE. Moreover, TRPC6 is highly expressed in breast carcinoma and is not correlated with estrogen receptor expression, tumour grade, or LNM.

Our results demonstrate that the OAG-activated cationic channels both in hBCE and MCF-7 cells share the same electrophysiological (lack of voltage dependence, and a similar reversal potential) and pharmacological properties (sensitivity to 2-APB, La^3+ ^and SK&F 96365). The TRPC candidates activated by OAG are limited to TRPC3, TRPC6, and TRPC7 [23,35]. TRPC6 is expressed at mRNA and protein levels both in MCF-7 and hBCE cells. Moreover, TRPC3 is also expressed in hBCE and its expression seems weak compared to that of the TRPC6 (Fig. 3A). However, the findings that hBCE cells express both TRPC3 and TRPC6, may indicate that the OAG-gated cationic channel(s) in theses cells are probably heterotetramultimers that include TRPC6/TRPC3.

We also extended our studies to examine TRPC6 expression at the protein levels in breast cancer tissues. No specific bands were obtained when we performed Western blot analyses: (i) on lysates from LNCaP, used as negative control [36], (ii) by omitting the primary antibody and (iii) by blocking the primary antibody with the TRPC6 peptide. In contrast, both tumour, MCF-7 and hBCE lysates produced a 97 kDa band corresponding to the expected size of full length TRPC6. Moreover, no other band was observed, suggesting that TRPC6γ splice variant is not translated into protein.

A growing number of studies demonstrate a close correlation between an overexpression of TRP channels particularly of the TRPV6 and TRPM8 families and the development of cancer [13,16]. However, little is known about the expression pattern of TRPC6 or its possible role in the development of cancer, and breast cancer in particular. To our knowledge, there is only one study which shows the involvement of the TRPC6 channels in the proliferation of epithelial human prostate cancer cells in primary culture [31]. Our results clearly show that in healthy breast tissues low levels of TRPC6 are detected in all cases determined. In contrast, the breast carcinoma tissue specimens revealed a significant overexpression of TRPC6. Moreover, TRPC6 are expressed and functional in the MCF-7 cell line and in hBCE. In vivo, the upregulation of TRPC6 was much more clearly demonstrated in cardiovascular pathologies such as hypertension, hypertrophy and increased endothelial permeability [27,29,30].

Few studies have compared the ionic channel expression with the tumour grade, LNM and receptor expression status. Indeed, GIRK1 was overexpressed in primary invasive breast carcinoma and correlate with LNM [37]. Pardo s group has found there were no correlations between Eag1 expression and age, grade and site of tumour of soft tissue sarcoma [38]. Similar results on Eag1 expression are found in colorectal cancer [39]. In line with these studies, we show that the TRPC6 protein levels are increased in breast tumour tissues but were not correlated either with tumour grade, ER or LNM.

Evidence indicates a crucial role for TRP channels in regulating both cell growth and cell death. Recently, it was reported that TRPV6 induced cell proliferation and took part in the resistance to apoptosis in the prostate human LNCaP cancer cells [40]. TRPC6 has been reported to be involved in primary epithelial prostate human cell proliferation induced by the α_1_-adrenergic receptors [31]. Breast cancer cells, including MCF-7 express G protein-coupled receptors including α1-adrenergic receptors [41]. We can thus speculate that the entry of Ca^2+ ^through TRPC6 channel may induce breast cancer cell proliferation in response to G protein-coupled receptor signalling. More studies are needed to determine the involvement of TRPC6 in breast cell proliferation.

## Conclusion

Our data demonstrate that TRPC6 is expressed and functional in breast cancer epithelial cells. Moreover, this channel is overexpressed in tumour tissues without any correlation with tumour grade, ER expression and lymph node metastasis.

## Competing interests

The authors declare that they have no competing interests.

## Availability & requirements

WebMaxC v2.1: 

## Authors' contributions

AG and YELH did the electrophysiological studies and MCF-7 cell culture. ID-D did the immunohistochemistry, the conventional PCR studies, and carried out the TRPC6 Western blots. NH and HK carried out the RNA extraction of the primary culture, cell line (MCF-7) and biopsy specimens. AA did the primary epithelial culture and corrected the manuscript. HS provided us with the human biopsies and allowed us to do the IHC in his laboratory. HO-A designed the studies and wrote the manuscript. All authors have read and approved the final manuscript

## Pre-publication history

The pre-publication history for this paper can be accessed here:


